# *Micronectagriseola* Horvath, 1899 - a new species of the family Corixidae (Heteroptera) for the fauna of the Middle Urals

**DOI:** 10.3897/BDJ.9.e71545

**Published:** 2021-09-13

**Authors:** Elena V Kanyukova, Vitaly A Stolbov, Sergey A Ivanov

**Affiliations:** 1 Zoological Museum оf Far Eastern Federal University, Vladivostok, Russia Zoological Museum оf Far Eastern Federal University Vladivostok Russia; 2 AquaBioSafe Laboratory, Tyumen State University, Tyumen, Russia AquaBioSafe Laboratory, Tyumen State University Tyumen Russia; 3 Tyumen State University, Tyumen, Russia Tyumen State University Tyumen Russia

**Keywords:** expansion of the range, Ob-Irtysh Basin, Russia, new data

## Abstract

**Background:**

Micronecta (Micronecta) griseola Horvath, 1899 is a representative of water bugs of the family Corixidae. It is expanding its range eastwards according to our observations in Russia. In the last decade, the species has become more common in Western Siberia.

**New information:**

The article presents the first records of *M.griseola* from the territory of the Middle Urals (Russia). The distribution of the species and the features of its biology are considered.

## Introduction

Water bugs of the Urals and Cis-Urals have not been sufficiently studied to date. Information on the finding of two species of the Corixidae family and two species of the Gerridae family in the Polar Urals is given by A.N. Kiritshenko (1916). In the summary of the fauna of aquatic insects in the vicinity of Sverdlovsk (Kolosov and Popov 1932), 11 species of the family Corixidae are mentioned. In a later work by A.N. Kiritshenko (1954), all the data of the previous faunists were summarised, their material rechecked and the erroneous indications of bedbugs for the Southern Urals eliminated. He listed 11 species of the Corixidae family for the Orenburg Region and adjacent areas of the middle reaches of the Ural River. In the generalising work of A.N. Zinovjeva (2013), based on the aquatic hemiptera fauna of the European North-East of Russia, partly affecting the Northern Urals, six species of Corixidae are given for the Urals. However, information on the species of the subfamily Micronectinae Jaczewski, 1924 in all these works is absent. This article presents the first records of Micronecta (Micronecta) griseola Horvath, 1899 from the Middle Urals (Sverdlovsk region).

In connection with the development of the national economy of the country in the 1920s , the employees of biological stations carried out an intensive study of water bodies and their inhabitants - the fodder base of fish. Extensive material on water bugs from the Volga, Oka, Kama and other water bodies, collected by A.L. Benning (Bening 1926, Bening 1928) and other hydrobiologists was identified by O.N. Sirotinina, who worked at the Volga biostation near Saratov. All specimens of *Micronecta* were determined by her as *M.minutissima* (Linnaeus, 1758), according to the European literature existing at that time. These data were repeated in our country by many faunists before the publication of the key to the European part of the USSR (Kerzhner and Jaczewski 1964). The keys to the genus *Micronecta* were compiled with the participation of the Polish specialist A. Wróblewski, who performed a systematic revision of the species of the genus and identified the features of species differences. He also studied the ecological and biological characteristics of *Micronecta* in Poland and other countries (Wroblewski 1958). A subsequent revision of the USSR fauna species from the ZIN collection by Wroblewski (Wroblewski 1962, Wroblewski 1963) showed that most of the previous records of *M.minutissima* from the European part of Russia are, in fact, *M.griseola* and *M.poweri* (Douglas and Scott, 1869), despite the fact that the real *M.minutissima* was reliably recorded from Russia only in Karelia and the Leningrad Region.

The number of erroneous indications also included the records of *M.minutissima* from the Southern Urals from the Orenburg Region (Bening 1926). Wroblewski (1963) identified the material and assigned it to *M.griseola*. Therefore, the indications of *M.minutissima* from the Urals - Udmurtia (Kargapoltseva et al. 2012) with reference to the works of Bening (1928), as well as the identification from Bashkiria (Boev and Bayanov 1989), need to be confirmed. A.A. Prokin (Papanin Institute for Biology of Inland Waters, RAS), by identifying material from Udmurtia, showed that *M.griseola* inhabits there (Kargapoltseva et al. 2013).

## Materials and methods

The collection of material in the Middle Urals was done in the eastern part of the Sverdlovsk Region in the spring-summer period of 2020. Small rivers of the Pyshma and Tura River Basins (Ob-Irtysh Basin) were investigated. The material was collected with a hydrobiological net, by catching water near the coast and mowing aquatic vegetation. The determination was made at the Hemiptera Department of the Laboratory of Insect Taxonomy of the Zoological Institute of the Russian Academy of Sciences (St. Petersburg), with the preparation of male parameres and comparison with the collection.

## Taxon treatments

### Micronecta (Micronecta) griseola

Horvath, 1899

84D76BB6-21E2-56C9-B86F-153896819731

#### Materials

- **Type status:**Other material. **Occurrence:** recordedBy: Vitaly Stolbov; lifeStage: 1 larva of the last instar; **Taxon:** kingdom: Animalia; phylum: Arthropoda; class: Insecta; order: Hemiptera; family: Corixidae; genus: Micronecta; subgenus: Micronecta; specificEpithet: *griseola*; scientificNameAuthorship: Horvath, 1899; **Location:** country: Russia; stateProvince: Sverdlovsk region; county: Slobodo-Turinsky district; locality: vicinity of the village Bobrovskoe; verbatimLocality: Bobrovka River, backwater, slow current, silty ground, depth 1 meter; verbatimElevation: 60 m a.s.l.; decimalLatitude: 57.498944; decimalLongitude: 64.148056; **Identification:** identifiedBy: Elena Kanyukova; **Event:** eventDate: 28.05.2020; **Record Level:** basisOfRecord: PreservedSpecimen
- **Type status:**Other material. **Occurrence:** recordedBy: Vitaly Stolbov; lifeStage: 2 males, 9 larvae of the last instar; **Taxon:** kingdom: Animalia; phylum: Arthropoda; class: Insecta; order: Hemiptera; family: Corixidae; genus: Micronecta; subgenus: Micronecta; specificEpithet: *griseola*; scientificNameAuthorship: Horvath, 1899; **Location:** country: Russia; stateProvince: Sverdlovsk region; county: Irbitsky district; locality: vicinity of the village Chernovskoe; verbatimLocality: Kirga River, depth more than a meter, fast current, sandy-silty soil, stones; verbatimElevation: 60 m a.s.l.; decimalLatitude: 57.609778; decimalLongitude: 63.382833; **Identification:** identifiedBy: Elena Kanyukova; **Event:** eventDate: 28.05.2020; **Record Level:** basisOfRecord: PreservedSpecimen
- **Type status:**Other material. **Occurrence:** recordedBy: Vitaly Stolbov; lifeStage: 1 larva of the last instar; **Taxon:** kingdom: Animalia; phylum: Arthropoda; class: Insecta; order: Hemiptera; family: Corixidae; genus: Micronecta; subgenus: Micronecta; specificEpithet: *griseola*; scientificNameAuthorship: Horvath, 1899; **Location:** country: Russia; stateProvince: Sverdlovsk region; county: Artyomovsky district; locality: vicinity of the village Antonovo; verbatimLocality: Borovaya River, depth 50 cm, fast current, muddy soil with separate stones, thread algae, *Sparganium* sp.; verbatimElevation: 95 m a.s.l.; decimalLatitude: 57.544000; decimalLongitude: 62.361556; **Identification:** identifiedBy: Elena Kanyukova; **Event:** eventDate: 28.05.2020; **Record Level:** basisOfRecord: PreservedSpecimen
- **Type status:**Other material. **Occurrence:** recordedBy: Vitaly Stolbov; lifeStage: 1 male, 252 1-2 instar larvae, 2 last instar larvae; **Taxon:** kingdom: Animalia; phylum: Arthropoda; class: Insecta; order: Hemiptera; family: Corixidae; genus: Micronecta; subgenus: Micronecta; specificEpithet: *griseola*; scientificNameAuthorship: Horvath, 1899; **Location:** country: Russia; stateProvince: Sverdlovsk region; county: Talitsky district; locality: Pripyshminskie Bory National Park; verbatimLocality: bypass channel of the Urga River, depth 30 cm, clay-sandy soil, fast current; verbatimElevation: 86 m a.s.l.; decimalLatitude: 56.976389; decimalLongitude: 63.681250; **Identification:** identifiedBy: Elena Kanyukova; **Event:** eventDate: 30.07.2020; **Record Level:** basisOfRecord: PreservedSpecimen

#### Diagnosis

##### Remarks

**Figure 1.**
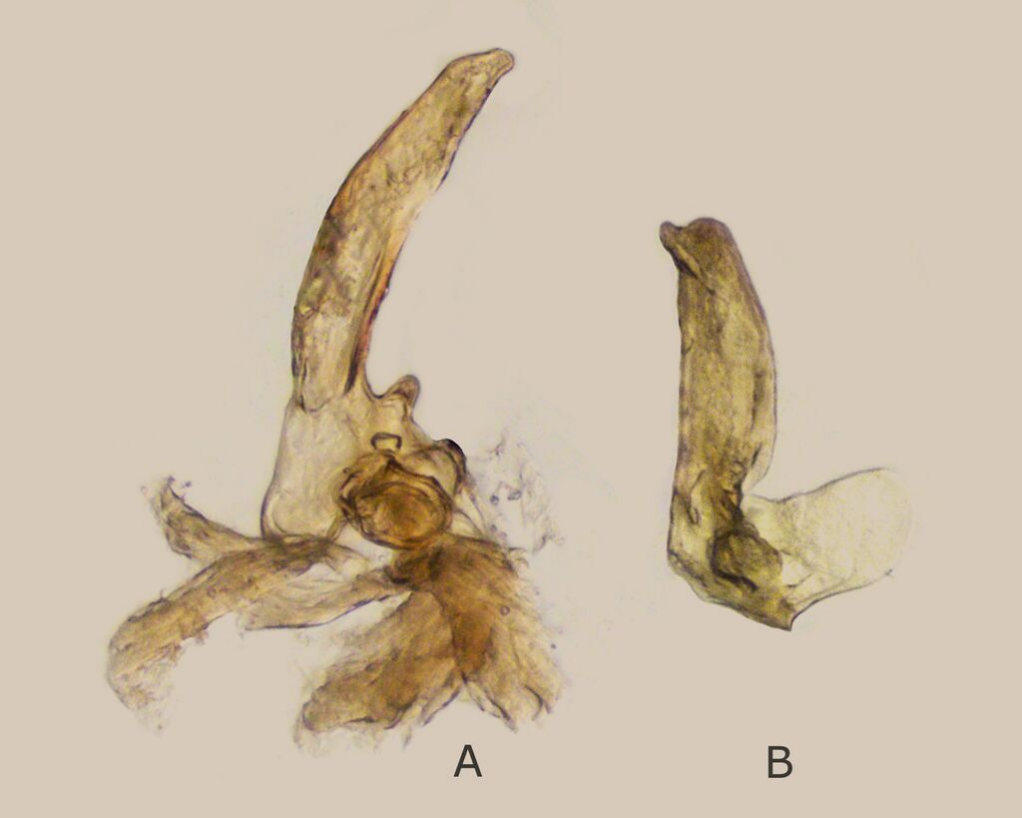
Parameres of male of *M.griseola* from Urga River **A** Right paramere; **B** Left paramere. Photos by D. Gapon.

The second author found *M.griseola* for the first time in the Sverdlovsk Region. Species of the genus *Micronecta* are the smallest representatives of the Corixidae family, the body length of the species ranges from 1.5 to 2.4 mm (Fig. 1). In *M.griseola*, as in other species of the genus, wing polymorphism is noted (Wroblewski 1958). Large and slender full-winged, flying forms with almost parallel elytral margins are rare. Short-winged non-flying forms predominate. They are distinguished by a shortened pronotum and an elliptical body shape, with elytra narrowed towards the apex and shortened wings (Kanyukova 2006).

#### Distribution

The range of distribution of the species is located in Western and Eastern Europe, from the British Isles (Brooke and Nau 2003) in the west to the Western Siberia and Central Asia in the east, from southern Finland in the north to Italy in the south (Kanyukova 2006). In the south of Russia, it is indicated from the North Caucasus (Shapovalov et al. 2014).The species was found in the Middle (our material) and South Urals (Wroblewski 1963). In the Ob Basin, the species was recorded from Novosibirsk (Belevich and Yurchenko 2012) and the south of Tyumen Regions (Kanyukova et al. 2019). It is known from the countries of Transcaucasia, Turkey, Kazakhstan (Wroblewski 1963), Kyrgyzstan, Tajikistan (Jansson 1986) and Northwest China (Kanyukova et al. 2016).

## Discussion

**Ecology and Biology**

**Figure 2.**
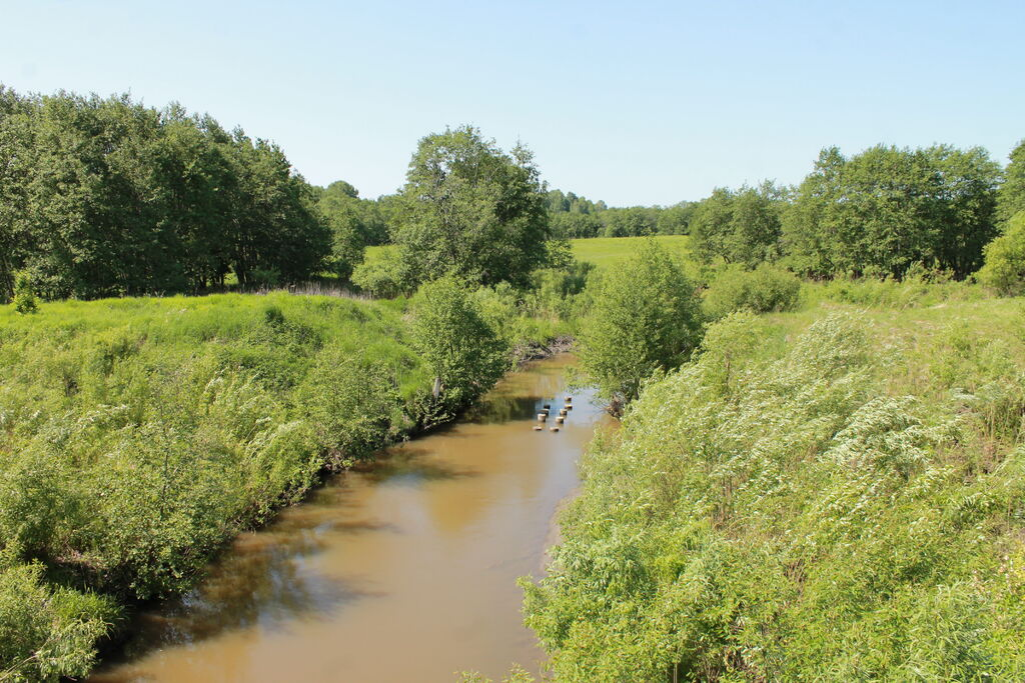
Habitat of *M.griseola* – Bobrovka River. Photo by V. Stolbov.

All of our finds of *M.griseola* were made in small rivers with different depths, flow rates and soil patterns; in three of the four rivers, higher aquatic vegetation was absent at the sampling site (Fig. 2). The studied rivers are located in the flat eastern part of the Sverdlovsk Region. The Rivers Bobrovka, Kirga and Borovaya are tributaries of the Tura River, the Urga River is a tributary of the Pyshma River. All rivers belong to the Ob-Irtysh Basin.

According to Wroblewski (1958), M.griseola lives in fresh flowing and stagnant water bodies and periodically occurring in large flocks off the coast. Most species of Corixidae hibernate in the adult stage, in contrast to the species of the subgenus Micronecta, which hibernate as translucent larvae of III and IV instars. Imagos disappear in late summer and autumn and they do not occur in early spring either. IV instar larvae often survive until spring. Micronects are polyvoltine and there are spring and summer generations (if the season is warm enough). In cold countries, one generation was recorded each summer (Kaiser 1966). The larvae overwintered in deep, non-freezing places in May-June, at temperatures above 15°C, reach the adult stage, giving rise to the imago of the 1st spring generation, which is later replaced by the 2nd generation. In the early stages, according to Wróblewski's observations, the larvae prefer great depths (Wroblewski 1958).

In a previous work (Kanyukova et al. 2019), we discussed the timing of the reproduction of this polyvoltinous species in the south of Tyumen Oblast, based on the dates of the collected material. It was suggested that adults of the first generation emerge in the first decade of June; adults of the second summer generation in the rivers of the Tyumen Region presumably appear from the second half of July.

Finds of older larvae and some imagos in the last ten days of May in the Middle Urals make it possible to attribute them to overwintered individuals and to consider this time as the date of the appearance of the first generation of adults of *M.griseola*. The date of collection of imagos and young larvae in the rivers of the Middle Urals on 30 July 2020, makes it possible to think that these adults belong to the second (summer) generation, which, after laying eggs, gives rise to wintering larvae. Although, like many insect species, *Micronecta*'s breeding times are extended during the summer and may overlap.

**Conclusion**

For the first time beyond the Urals, in Western Siberia, *M.griseola* was recorded in the Novosibirsk Region in 2010. All records from the southwest of Western Siberia in the Tyumen Region were made by authors within several years after 2017. Findings of *M.griseola* in large numbers in four rivers of the Sverdlovsk Region in 2020 suggest that this species has been actively spreading in recent years over the territory of the West Siberian Plain (Ob-Irtysh Basin).

In Europe, in recent decades, data on new finds of *M.griseola* in different countries have also been published (Aukema et al. 2000, Brooke and Nau 2003, Cuppen and Nelson 2007), which indicates the movement of the species range in a westerly direction.

The expansion of the ranges of a number of species in recent years has been noted in many regions, including Siberia. Often, in the case of terrestrial animal and plant species, this phenomenon is associated with global climate change (Stolbov et al. 2016). The situation of *M.griseola* is also evidence of the ecological plasticity of the species. Often, the expansion of the ranges of aquatic organisms is associated with human economic activities - unintentional introduction and transformation of water bodies (Kurina 2020). Identifying the reasons for the expansion of the *M.griseola* range requires additional research.

## Supplementary Material

XML Treatment for Micronecta (Micronecta) griseola
